# The long-term effect of acupuncture for patients with knee osteoarthritis: Study protocol for a randomized controlled trial

**DOI:** 10.1097/MD.0000000000022599

**Published:** 2020-10-16

**Authors:** Nan Wu, Jin Huang, Xuguang Yang, Jian Guo, Feilai Liu, Yujing Gu, Yongtao Liu, Zhenhua Zhang, Shuai Yin, Xiaodong Feng

**Affiliations:** a School of Rehabilitation Medicine, Henan University of Chinese Medicine; b The Rehabilitation Center, First Affiliated Hospital of Henan University of Chinese Medicine.

**Keywords:** *deqi*, knee osteoarthritis (KOA), long-term effect of acupuncture, randomized controlled trial

## Abstract

**Introduction:**

Whether there is the long-term effect of acupuncture on patients with knee osteoarthritis (KOA) or not is controversial. According to the basic theory of traditional acupuncture, *deqi* is the key to the efficacy of acupuncture. This randomized controlled trial aims to evaluate the existence of long-term effects caused by *deqi* in patients with KOA.

**Methods and analysis:**

A three-armed, parallel-design, randomized controlled trial is underway in China.108 KOA patients recruited by the rehabilitation center of the First Affiliated Hospital of Henan University of Traditional Chinese Medicine will be randomly assigned to the acupuncture with *deqi* group (A group), the acupuncture without *deqi* group (B group) and the waiting-list group (C group). Each patient will receive 5 30-minute sessions per week for 4 consecutive weeks and rest for 2 days between treatments, and undergo a 20-week follow-up. The primary outcome is the Western Ontario and McMaster Universities Osteoarthritis index (WOMAC score). The secondary outcomes include Western Ontario and McMaster Universities Osteoarthritis index (WOMAC score), Knee Injury and Osteoarthritis Outcome Score (KOOS), arthritis quality of life measurement scale simplified scale (AIMS2-SF), emotional monitoring and expectation scale. The pain visual analogue scale (VAS) and the Chinese version of modified Massachusetts General Hospital Acupuncture Sensation Scale (C-MMASS) will be used to evaluate the *deqi* sensation after each acupuncture treatment. At the same time, adverse events (AEs) occurred in the whole process will be recorded and analyzed. We will perform an intention-to-treat analysis and protocol (PP) analysis to statistically analyze the results of the trial.

**Discussion:**

This trial will be useful to study the long-term effect of acupuncture and the influence of the *deqi* sensation on the long-term in the treatment of KOA, and to provide a clinical basis for treatment of patients with mild to moderate knee osteoarthritis in clinic.

**Trial registration:**

Chinese Clinical Trial Registry, IDF: ChiCTR2000029291. Registered on January 21, 2020.

## 1 Introduction

Knee osteoarthritis, also known as degenerative arthritis or osteoarthritis, is a common clinical disease and high incidence, and is one of the most common diseases in middle-aged and elderly people.^[1]^ Chronic pain is a common clinical symptom of KOA,^[2]^ which seriously reduces the quality of life of patients and is likely to cause other life dysfunction. In severe cases, it even loses joint function and seriously affects the normal life and work of patients. It has become a public health issue of increasing concern in an aging society around the world. Among the reasons for the loss of labor in Europe and the United States, KOA is ranked 4th in women and 8th in men^[3]^ respectively. Among them, over 9 million adults in the United States have KOA, and this prevalence is increasing year by year.^[4]^ A study in China showed that the prevalence of KOA was 33.3% in people over 40 years old, 39.4% in those over 60 years old, and 45.7% in those over 70 years old.^[5]^ But there is no especially effective treatment for KOA. Approximately one-third of direct osteoarthritis expenditures were from medications, mainly for pain-related agents.^[6]^ Currently, most of the clinically targeted drugs for KOA are non-steroidal anti-inflammatory drugs (NSAIDs) and COX-2 selective agents, they are associated with an increased risk of gastrointestinal bleeding and cardiovascular events.^[7]^ And according to multiple research studies, acupuncture has a good therapeutic efficacy on bone impediments, including KOA.^[8–10]^ A number of studies have proven that acupuncture is very effective in the treatment of KOA.^[11,12]^ One systematic review evaluated that acupuncture was shown to be effective for pain control in KOA in sham-controlled RCTs.^[11]^ In the Cochrane review, researchers found that acupuncture had statistically significant and clinically relevant benefits in the treatment of patients with peripheral OA in the waiting list-controlled trials.^[12]^ As a result, considering the short-term analgesic effect and worrying side effects of drugs, more and more people are choosing supplemental replacement therapy with better sustained analgesic effect to treat KOA, and acupuncture is an important tool.

*Deqi*, the ancient name is “*qi* arrival”, also known as “needle sense”, refers to the needle into the acupoints a certain depth, after applying a certain needle-handling method, so that the acupuncture site obtains the induction of *qi*. *Deqi* is the key to the effectiveness of acupuncture and is the basis for the long-term analgesic effect of acupuncture. The long-term effect is the therapeutic effect that still exists after the completion of acupuncture treatment, and is the accumulation and continuation of the immediate effect of acupuncture. The long-term effect has been reported in trials of acupuncture for treating chronic tension-type headache,^[13]^ migraine,^[14]^ primary insomnia,^[15]^ tinnitus,^[16]^ etc. However, the long-term effect of acupuncture on KOA has not been confirmed, so we design a randomized controlled trial (RCT) with a treatment period of 4 weeks and an observation period of 20 weeks to investigate and evaluate the long-term effect of acupuncture for KOA.

## 2 Methods

### 2.1 Study design and registration

**Figure 1:**
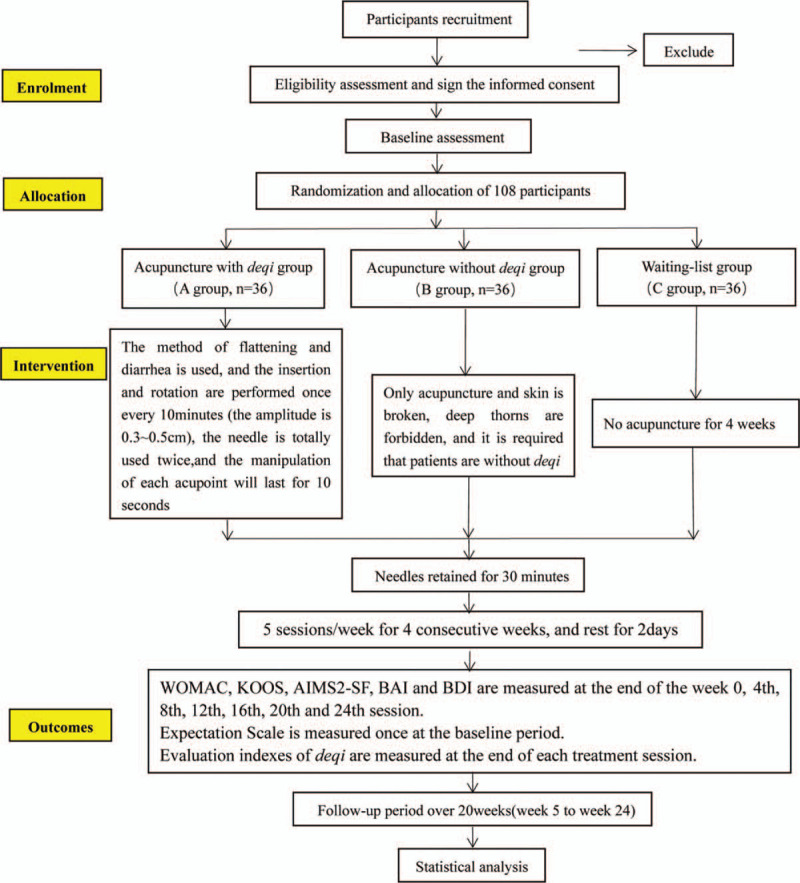
Flow chart of the trial.

This is a parallel-design, randomized controlled trial comparing the therapeutic outcomes of the acupuncture with *deqi* group, the acupuncture without *deqi* group and the waiting-list group. And this trial belongs to the clinical randomized controlled study, which aims to compare the long-term effect of acupuncture treatment among the 3 groups. This trial will be carried out at the Rehabilitation Center of the First Affiliated Hospital of Henan University of Traditional Chinese Medicine, Zhengzhou, Henan, China. The procedure will last for 24 weeks, including a 4-week treatment and a follow-up period of 20 weeks. Patients in *deqi* group and without *deqi* group will receive 20 sessions of acupuncture during the 4-week treatment. The Figure 1 shows the study design in the flowchart, and the Figure 3 illustrates the time schedule of enrolment, interventions, assessments, and visits of participants. The reporting of this trial is conducted according to the Standard Protocol Items: Recommendations for Intervention Trials (SPIRIT) guidelines (Additional file 1).

This protocol has been registered in the Chinese Clinical Trial Registry on January 21, 2020, http://www.chictr.org.cn/edit.aspx?pid=38787&htm=4. And the registration number is ChiCTR2000029291.

### 2.2 Participants

#### 2.2.1 Inclusion criteria

① male or female, aged 45 to 75 years; ② Diagnosis of knee osteoarthritis according to the American College of Rheumatology (ACR) criteria;^[17]^ ③ Radiologic confirmation of knee osteoarthritis (Kellgren–Lawrence grade I or III); ④ the average daily pain over 40 points (on a 0-to 100-point scale); ⑤ written consent to participate in the trial.

#### 2.2.2 Exclusion criteria

Subjects will be excluded if they meet any of the following criteria: ① are unable to walk; ② have a serious infection of the knee; ③ have suspected tears in any ligaments or menisci or acute inflammation of the synovial capsule; ④ have a history of trauma, ligament damage, fracture, or surgery on the knee(s) within 6 months, causing pain or functional problems (history of knee replacement will be excluded); ⑤ have a history of local tumor/malignancy at the knee; ⑥ have physical or laboratory findings indicating infection, presence of autoimmune disease or inflammatory arthritis; ⑦ have knee pain caused by radiculopathy/herniation of an intervertebral disc; ⑧ have end-stage diseases or other suspected severe conditions such as deep vein thrombosis of the lower limb, oedema related to cancer or cancer treatment, severe blood coagulation disorders, uncontrolled systemic arterial hypertension and severe diabetes; ⑨ have a history of prolotherapy, hyaluronic acid injections or corticosteroids injections within 3 months; 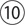 have received acupuncture, electro-acupuncture, Tui-na therapy, massage, or physiotherapy 8 weeks prior to enrolment in the trial; 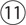 have severe pain in other regions; 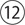 have severe mental disorder(s); 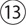 are oversensitive to needles; and 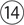 are insensitive to pain due to advanced diabetes, neuropathy or use of strong painkillers.

### 2.3 Sample size

PASS was used for sample size determination. According to the previous study, WOMAC Function Score of KOA patients decreased by 12.1 points after acupuncture, by 9.4 points after sham acupuncture and 5.6 points after education, while WOMAC pain Score of KOA patients decreased by 3.6 points after acupuncture, by 2.6 points after sham acupuncture and 1.5 points after education.^[18]^ Based on the previous study and our clinical experience, we anticipated a reduction of WOMAC total score by 17 points after acupuncture with the *deqi* sensation, a reduction by 13 points after acupuncture without the *deqi* sensation and a reduction by 8 points in the wait-list control. With α = 0.05, 1 – β = 0.9, and a standard deviation of 10, we need at least 99 participants in total. Considering a dropout rate of 10%, a total of 108 participants will be included in this trial.

### 2.4 Randomization

Patients who meet the inclusion criteria will be randomly assigned to 3 groups of the trial in a ratio of 1:1:1 by a third party using simple concealed randomization to avoid possible selection bias. And the random assignment protocol will be generated by the third party who will not participate in the trial and use the PROCPLAN process of the SAS package. At 5 to 10 minutes prior to the acupuncture treatment, the acupuncturist will inform the manager of the randomization program about the patient number and name via telephone; the acupuncturist will then be informed of the patients randomization number and treatment group. The randomization information will be concealed in the server until the end of the trial.

### 2.5 Blinding

The acupuncturist and patients will not be blinded because of the nature of the intervention. However, it is feasible to conceal the researchers, statisticians and the outcome assessors. The outcome assessor and data analyst will be blinded to group assignments. Unblinding will not be done until the completion of data analysis, and after the data analysis, we will have blinded interpretation of the study results to minimize misleading data interpretation additional file 1.

### 2.6 Researchers

The treatment will be performed by the same licensed acupuncturist with more than 6 years experience. In addition, 2 supervisory data collectors and analysts who do not know the random assignment protocol will monitor the whole experiment. People who are responsible for recruiting participants, researchers, outcome assessors, and the statistician in the trial will be trained before the trial strictly.

### 2.7 Recruitment

The patients will be recruited mainly through the outpatient and inpatient areas of the rehabilitation center of the First Affiliated Hospital of Henan University of Traditional Chinese Medicine, and we will use the Internet for online recruitment. The poster will briefly describe the trial and provide treatment details and contact details to eligible participants, and contact information. Participants who are interested in participating will be able to contact the researcher directly.

We will provide the potential participants with a detailed description of the benefits of the trial and acupuncture and the risks that may exist. If they decide to participate, they will be asked to sign the informed consent. Then, they will be included in this trial for randomization. If a participant withdraws from the trial, the reasons for withdrawal will be recorded.

In order to improve the patients compliance in the trial, we will strengthen the doctor-patient communication and the patient's health education timely, to increase the patients’ trust in members of the research group.

### 2.8 Safety assessment

Patient safety will be assessed in the whole process to avoid adverse events (AEs). All unpremeditated attacks and unexpected effects will be recorded on the AE Report Form. Treatment-related AEs include pain, haematoma, localized infection, broken needle, fainting, nausea, headache, dizziness, insomnia, or vomiting during or after treatment. Any serious adverse events (SAEs) will be immediately reported to the principal investigator and the Medical Ethics Committee within 24 hours. A primary investigation and follow-up monitoring will be performed if AEs are reported.

## 3 Interventions

There are 3 groups in this trial, which are group A (*deqi* group), group B (without *deqi* group), and group C (waiting-list group).

**Figure 2:**
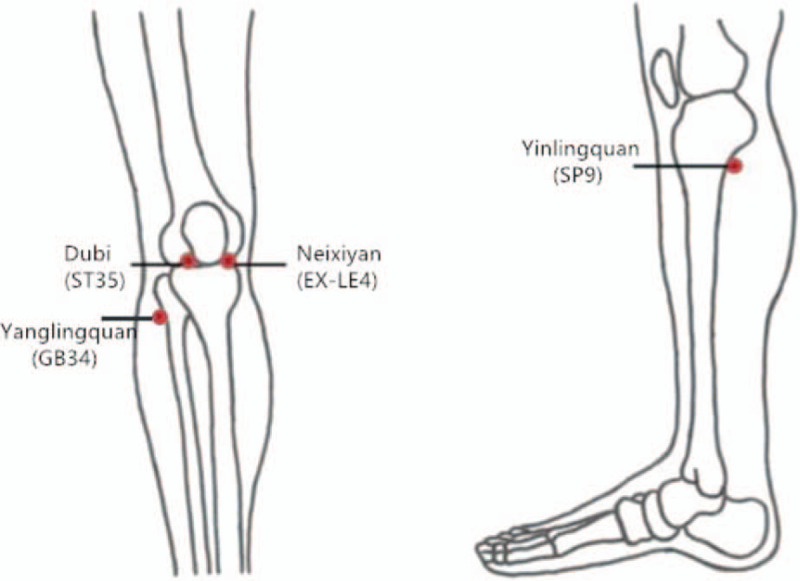
The picture of acupuncture points.

The acupoints (Fig. 2) used in the trial include *Yanglingquan* (GB34), *Yinlingquan* (SP9), *Dubi* (ST35), and *Neixiyan* (EX-LE4). At the same time, depending on the patient's condition, 2 local acupuncture points can be added.

The acupuncture operation will be performed by the same licensed acupuncturist with more than 6 years experience, and will be trained in the clinical operation organized by the research group. Acupuncture treatment once a day, 5 sessions per week for 4 consecutive weeks, and rest for 2 days between treatments.

### 3.1 Acupuncture with the deqi group

The specific operation method is as follows: after the patients in group A maintain a comfortable position, and the method of flattening and diarrhea is adopted. Then disinfect the skin with 75% alcohol, acupuncturist uses a sterile needle (0.25 mm in diameter, 40 mm in length, *Huatuo*, Suzhou, China) to the acupuncture points. Then, the needle is twisted between 90 and 180, lifted and thrusted in an even amplitude between 0.3 cm to 0.5 cm, 60 times to 90 times per minute. After the *deqi* sensation (including soreness, numbness, distention, and heaviness) is obtained, the needle will be retained for 30 minutes in each session and manipulated every 10 minutes with intermittent stimulation for maintaining the *deqi* sensation. The needle is totally used twice and the manipulation of each acupoint will last for 10 seconds. Table 1.

**Table 1:**
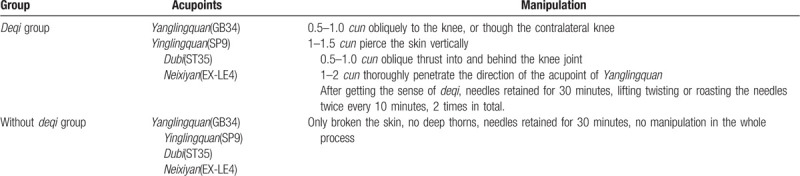
Detailed information on acupuncture at each acupuncture point.

### 3.2 Acupuncture without the deqi group

Subjects in the acupuncture without *deqi* group will undergo the same procedures as those in the acupuncture with *deqi* group. The needle depth is 1–2 mm and the needle will be directly retained for 30 minutes without any manipulation in the whole procedure to avoid the *deqi* sensation as much as possible (Table 1).

### 3.3 Waiting-list group

Patients will not receive acupuncture treatment during the trial. These patients will be treated by acupuncture with *deqi* after finishing the trial.

If the patient has pain, bleeding, hematoma or other severe discomfort, the acupuncture treatment should be interrupted and treated immediately. During acupuncture treatment and follow-up, patients are prohibited from using drugs that treat knee osteoarthritis and relieve mood. If necessary, the details of the medication used and dosage of medication used should be recorded in the case report forms (CRFs).

## 4 Outcome assessments

If only one knee is affected, the assessment of the outcomes will relate to this knee. If the patient has 2 affected knees of which only one meets the ACR criterion, only this knee will be evaluated. In the case that both knees are affected in accordance with the inclusion criteria (ACR), the more painful knee will be randomly chosen for evaluation.

### 4.1 Primary outcome

We will use the Western Ontario and McMaster Universities Osteoarthritis Index (WOMAC) as the primary outcome measure.^[19]^ It consists of 24 items assessing the patients with KOA pain (5 items), stiffness (2 items) and physical function (17 items). The score ranges for the pain, stiffness and physical function subscales are, respectively, 0–50, 0–20 and 0–170, resulting in a total range of 0–240. Higher scores indicate more severe symptoms or physical disability. The evaluation will be performed at week 16 after randomization.

### 4.2 Secondary outcome

#### 4.2.1 Western Ontario and Mcmaster Universities Osteoarthritis Index (WOMAC)

The evaluation will be also performed at week 0, 4th, 8th, 12th, 20th, and 24th after randomization.

#### 4.2.2 Knee injury and osteoarthritis outcome score (KOOS)^[20]^

**Figure 3:**
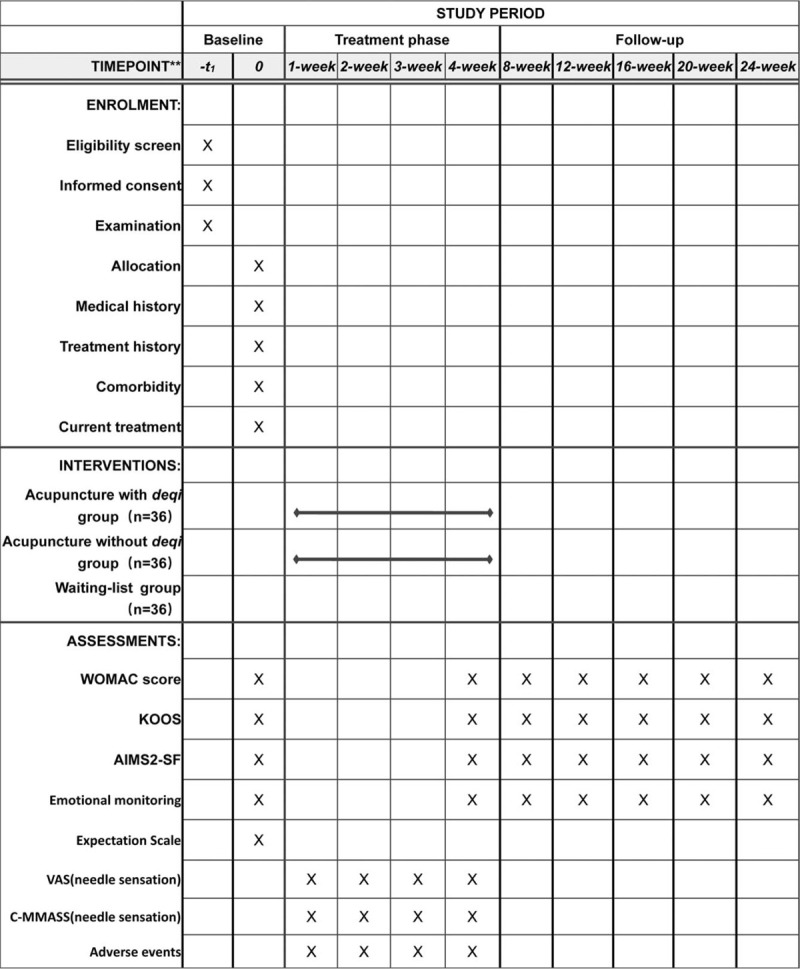
Study design schedule.

The KOOS (Fig. 3) is self-assessed by the patient. The scoring system consists of 5 sub-domains with a total of 42 individual items. The 5 sub-domains are symptoms (7 items), pain (9 items), activities of daily living (17 items), entertainment and motor function (5 items), and quality of life associated with the knee joint (4 items). For each sub-scale, the outcome score will be normalized, where 0 indicates no symptoms and 10 indicates the most extreme symptoms. The evaluation will be performed at week 0, 4th, 8th, 12th, 16th, 20th, and 24th after randomization.

#### 4.2.3 Arthritis quality of life measurement scale simplified scale (AIMS2-SF)^[21]^

Quality of life assessment using the AIMS2-SF Chinese version of the Arthritis Quality of Life Measurement Scale Simplified Scale by the Fudan University School of Public Health. The scale consists of 5 dimensions, 26 entries, of which 12 items of body (activity, walking, hand, arm, self-care), 3 items of symptoms (pain caused by arthritis), 5 items of emotion (the emotional impact of arthritis), social 4 items (social activities and social support) and work 2 items, because this test for KOA patients, the body dimension hand and arm a total of 5 items removed, a total of 21 items. All entries are scored on a scale of 1 to 5, and the total score is cumulatively represented by a score of 5 dimensions. The higher the score, the better the quality of life. The evaluation will be performed at week 0, 4th, 8th, 12th, 16th, 20th, and 24th after randomization.

#### 4.2.4 Emotional monitoring

Mood changes, such as depression and anxiety, are common concomitant symptoms of KOA. The Beck Anxiety Inventory (BAI),^[22]^ and the Beck Depression Inventory (BDI)^[23]^ will be used in this trial to evaluate the emotional changes of patients in order to monitor the impact of emotional changes on the condition of KOA. The evaluation will be performed at week 0, 4th, 8th, 12th, 16th, 20th, and 24th after randomization.

#### 4.2.5 Expectation scale

The treatment expectancy of participants will be evaluated by the Treatment Expectancy Questionnaire (1 = not at all, 5 = somewhat and 9 = very much), which has been transformed and translated into Chinese from the “Credibility and Expectancy Questionnaire”.^[24]^ Participants will be asked “How much do you feel acupuncture therapy will help to reduce your symptoms?” at the first visit.

### 4.3 Evaluation indexes of *deq*i

Before the acupuncture treatment, each patient will be introduced with the concept of *deqi* and the sensation of *deqi*. Participants in the acupuncture with the *deqi* group and acupuncture without the *deqi* group will be asked to fill the needle sensation evaluation form after receiving each acupuncture treatment.

#### 4.3.1 The pain visual analogue scale (VAS)^[25]^

The VAS scale is used to quantitatively evaluate the sensation of aspiration after the acupuncture treatment. The intensities of these sensations are evaluated on a 0 to 100 mm VAS, where 0 represents “none” and 100 represents “intolerable strong sense”. The participant will mark a point on the horizontal line according to the feeling of self, indicating the degree of pain. In addition, if the scale does not fully describe the acupuncture sensation experienced by the subject, the subject can add their sensation in a blank line below the scale.

#### 4.3.2 The Chinese version of modified Massachusetts General Hospital acupuncture Sensation Scale (C-MMASS)^[26]^

Each patient will be required to complete a C-MMASS form at each end of the acupuncture treatment in order to assess the intensity of the patient's 11 senses: soreness, aching, deep pressure, heaviness, fullness/distension, tingling, numbness, dull pain, warmth, cold, and throbbing.

In order to ensure uniform standards for the trial, the researcher, data collector and statistician responsible for the recruitment must undergo rigorous training before the trial and understand the purpose, contents and quality control of the trial. Detailed evaluation time points are provided in the Figure 3.

### 4.4 Data management

Regular monitoring that will be clarified in a standard operating procedure will be conducted at each site to ensure good data quality. The data of the trial needs to be recorded in the CRFs that prepared in both paper and electronic versions. Monitors will audit data every 3 months. During the trial, only the data evaluator can access the CRFs and enter the data in the electronic CRFs. Both the acupuncturist and the data analyst will have no access to these data during the evaluating process. When the trial is completed, the database will be locked by the data management team, after which the researchers can no longer modify the data. Both paper files and electronic documents will be preserved for at least 5 years after publication. If readers and reviewers have any questions, they can contact the corresponding author for access to the original data. Interim analyses will not be undertaken at any time during the whole period of study.

### 4.5 Ethics

This study is approved by the Ethics Committee of the First Affiliated Hospital of Henan University of Traditional Chinese Medicine (Ethics Reference No: 2019HL-020–01), and registered in the Chinese Clinical Trial Registry on January 21, 2020, ChiCTR2000029291, http://www.chictr.org.cn/edit.aspx?pid=38787&htm=4. The trial follows strictly the guidelines of the Declaration of Helsinki (Version 2000). Only participates who signed the informed consent form will be included.

### 4.6 Statistical analysis

In this trial, we will implement the intention-to-treat analysis and per-protocol (PP) analysis. The intention-to-treat analysis will be used for all allocated participants in the baseline. This trial focuses on the long-term effect of acupuncture, and the observed data will be used as the primary analysis. Primary analysis will be based on intention-to-treat population, including participants with at least 1 assessment of the primary outcome and 1 acupuncture session. Per protocol (PP) analysis will be performed to test the robustness of the primary analysis. PP population will include participants receiving at least 12 sessions of acupuncture and be assessed at week 16.

The demographic and baseline characteristics among 3 groups will be compared using one-way analysis of variance (ANOVA). A χ^2^ test will be used for categorical variables. Due to the occurrence of self-healing tendency in KOA, it is important to testify the existence of the long-term effect of acupuncture effect. The clinical outcomes will be compared among the acupuncture with the *deqi* group, acupuncture without the *deqi* group and the waiting treatment group at every time points (week 0, 4th, 8th, 12th, 16th, 20th, and 24th after randomization) by using the repeated measures ANOVA (3 independent groups with repeated measures).

Clinical outcomes will be analyzed using SPSS 22.0 statistics software (IBM SPSS Statistics, IBM Corp, Somers, NY, USA). Data on Skewed distribution will be normalized. Means, standard deviations, and 95% confidence intervals (CIs) will be used to describe the continuous data. All statistical hypotheses are two-sided tests, and the significance test level α = 0.05, giving the test statistic and its corresponding *P* value, with *P* ≤ .05 as statistically significant. The data will be analyzed by statisticians who are blinded to the test settings. All confidence intervals will be two-sided 95% intervals among the 3 groups (group A, group B and group C).

## 5 Discussion

According to the characteristics of KOA pain, it is a category of “knee cramps”. Chronic pain is a common clinical symptom of KOA, which directly leads to the decrease of functional activities and quality of life.^[2]^ The main treatment goals of KOA are to relieve or eliminate pain, improve joint function, prevent and delay the progression of the disease, and improve the quality of life of patients,^[27]^ but there is no good treatments currently. As a representative of traditional Chinese medicine, acupuncture has a remarkable curative effect on KOA,^[28,29]^ and the long-term effect of acupuncture which is an important feature of acupuncture analgesia has been confirmed in many clinical trials.^[30–34]^ However, KOA is a chronic joint disease, and the long-term effect is very important for the evaluation of the treatment of acupuncture on KOA, but whether there is a long-term effect on KOA requires further studies.

“The quicker the *qi* arrives, the faster the acupuncture effect acquires” (“*Biao You Fu*,” a Poetic Prose on the Elucidation of Acupuncture). *Deqi* is not only the key to acupuncture treatment, but also a good entry point to study the effect of acupuncture on the long-term analgesia effect of KOA. Modern clinical trials have considered that *deqi* plays an essential role in the process of achieving the acupuncture effect.^[26,35–37]^ For example, Xiong et al^[35]^ found that in the treatment of primary dysmenorrhea by acupuncture, the pain level of the *deqi* group was significantly improved after acupuncture treatment, which led them to conclude that *deqi* plays a decisive role in the therapeutic effect of acupuncture. In a systematic review of chronic pain in the knee, Wu WF et al^[36]^ found that acupuncture at *Quch*i Point (LI11) for the treatment of primary high acupoint pressure showed that the antihypertensive effect of the non-*deqi* group was significantly lower than that of the *deqi* group.

The long-term effect of acupuncture which is an important feature of acupuncture treatment after cessation^[38]^ refers to the acupuncture effect that persists after the end of acupuncture treatment. Compared with immediate effect, the long-term effect is stronger, wider, more lasting and can be accumulated.^[39]^ Many studies have shown that the long-term effect is an important part of acupuncture that is different from placebo effect, and it is an important focus for improving the efficacy of acupuncture. It has been recorded in a lot of ancient Chinese medical books and reported in many kinds of clinical and animal trials. For example, acupuncture treatment for chronic tension-type headache,^[13]^ migraine,^[14]^ primary insomnia,^[15]^ tinnitus,^[16]^ chronic knee pain,^[40]^ rats with cerebral ischemia and reperfusion,^[41]^ etc. These studies have confirmed that the long-term effect is an important feature of acupuncture analgesia. However, the long-term effect is not clear about the efficacy of KOA. Therefore, in order to demonstrate the effectiveness of the *deqi* sensation on the long-term acupuncture effect in patients with knee osteoarthritis, we design a randomized controlled trial with a treatment period of 4 weeks and an observation period of 20 weeks to investigate and evaluate the long-term effect of acupuncture for KOA.

The randomized controlled study design is an in-depth study of reliable methods based on the long-term analgesic effect of acupuncture. At the same time, it is a randomized controlled study that is an important source of high-quality evidence-based medical evidence for acupuncture. In the trial, we take acupuncture without *deqi* group as the control group. It is often regarded as a sham control group, because its acupuncture depth is only broken and does not reach the anatomical layer of the acupuncture point. Studies^[42–44]^ have shown that the placebo effect of sham acupuncture is more pronounced than drug-based placebo, especially for subjective outcomes and pain management. Therefore, our study has set a sham acupuncture group to eliminate the effects of placebo effects and patients’ expectations. And in the idea of acupuncture treatment of knee osteoarthritis, local acupoints have been used since ancient times. “*Su Wen*” says, local *Ashi* Point for the treatment of knee pain. A number of modern clinical studies^[44,45]^ have shown that local acupoints are widely used in the treatment of KOA by acupuncture, and the curative effect is remarkable. Some researchers^[45]^ used data mining to analyze the relevant literature of modern acupuncture treatment for KOA, and found that *Dubi*, *Neixiyan* and *Yanglingquan* were the top 3 acupoints with the high frequency. Another study^[46]^ shows that *Ashi* point is also one of the high-frequency acupoints. Thus, this study will select *Dubi*, *Neixiyan* and *Yanglingquan*, *Yinlingquan* as the acupuncture points. At the same time, depending on the patient's condition, 2 local acupuncture points can be added.

In order to improve the reliability of the study data, we carry out quality control from the following aspects:

1. Recruitment of middle-aged and elderly patients with mild to moderate recurrent knee pain in the past month to avoid the impact of other diseases on pain assessment;
2. In order to alleviate patients doubts about different interventions, patients in each group will be treated in different rooms, and patients in each group will be forbidden to talk.
3. The acupuncturist is a doctor with more than 6 years of work experience and a medical practitioner certificate. Relevant personnel and data evaluators responsible for recruiting patients are required to undergo rigorous training before the study to ensure that all experimental procedures have uniform standards and avoid observational bias.
4. Emotion monitoring. During the whole study, not only the emotional changes of patients will be evaluated on time by the emotion monitoring scales, but also the emotional changes of each patient should be paid close attention in order to eliminate the influence of emotional changes on the disease.

Of course, this study also has some shortcomings, mainly reflected in the following 2 aspects:

1. Because this study has a long observation period, it will result in a higher rate of abscission, which will affect the results of the study. Therefore, we should maintain a good doctor-patient relationship with patients during the study, and the part of the scales involved in this study are self-rating scale, which can be carried out subjectively by patients to better improve the quality of studys data;
2. The patients enrolled in this study are limited to mild to moderate KOA patients, so the results of this study have some limitations, they may only be applicable to mild to moderate KOA individuals.

Most domestic and international medical treatment guidelines recommend acupuncture for knee osteoarthritis, but the long-term effect of acupuncture for KOA is not fully defined. Therefore, this study designs a clinical study with a treatment period of 4 weeks and a follow-up period of 20 weeks, aiming to study the long-term effect of acupuncture and the influence of the *deqi* sensation on the long-term in the treatment of KOA, and to provide a clinical basis for treatment of patients with mild to moderate knee osteoarthritis in clinic.

## 6 Trial status

Protocol version number V1.0, January 21, 2020. The trial is currently in the participant recruitment stage. The trial began recruitment on March 1, 2020 and is expected to complete on December 31, 2021.

## Acknowledgments

We are grateful to Ph.D. Ruirui Sun, a teacher of acupuncture and massage at Chengdu University of Traditional Chinese Medicine (TCM), for helping us design the thesis and English language and grammar editing.

## Author contributions

**Conceptualization:** Shuai Yin, Xiaodong Feng.

**Data curation:** Nan Wu, Zhenhua Zhang.

**Methodology:** Xuguang Yang, Shuai Yin.

**Project administration:** Jin Huang, Jian Guo.

**Supervision:** Yujing Gu.

**Validation:** Yongtao Liu.

**Writing – original draft:** Jin Huang.

**Writing – review & editing:** Feilai Liu, Shuai Yin, Xiaodong Feng.
